# Neutrophils Turn Plasma Proteins into Weapons against HIV-1

**DOI:** 10.1371/journal.pone.0066073

**Published:** 2013-06-26

**Authors:** Cornelia Speth, Martin F. Brodde, Magdalena Hagleitner, Günter Rambach, Hugo Van Aken, Manfred Dierich, Beate E. Kehrel

**Affiliations:** 1 Division of Hygiene and Medical Microbiology, Innsbruck Medical University, Innsbruck, Austria; 2 Klinik für Anästhesiologie, operative Intensivmedizin und Schmerztherapie, Universitätsklinikum Münster, Westfälische Wilhelms Universität Münster, Münster, Germany; Scripps Research Institute, United States of America

## Abstract

As a consequence of innate immune activation granulocytes and macrophages produce hypochlorite/hypochlorous acid (HOCl) via secretion of myeloperoxidase (MPO) to the outside of the cells, where HOCl immediately reacts with proteins. Most proteins that become altered by this system do not belong to the invading microorganism but to the host. While there is no doubt that the myeloperoxidase system is capable of directly inactivating HIV-1, we hypothesized that it may have an additional indirect mode of action. We show in this article that HOCl is able to chemically alter proteins and thus turn them into Idea-Ps (Idea-P = immune defence-altered protein), potent amyloid-like and SH-groups capturing antiviral weapons against HIV-1. HOCl-altered plasma proteins (Idea-PP) have the capacity to bind efficiently and with high affinity to the HIV-1 envelope protein gp120, and to its receptor CD4 as well as to the protein disulfide isomerase (PDI). Idea-PP was able to inhibit viral infection and replication in a cell culture system as shown by reduced number of infected cells and of syncytia, resulting in reduction of viral capsid protein p24 in the culture supernatant. The unmodified plasma protein fraction had no effect. HOCl-altered isolated proteins antithrombin III and human serum albumin, taken as representative examples of the whole pool of plasma proteins, were both able to exert the same activity of binding to gp120 and inhibition of viral proliferation. These data offer an opportunity to improve the understanding of the intricacies of host-pathogen interactions and allow the generation of the following hypothetical scheme: natural immune defense mechanisms generate by posttranslational modification of plasma proteins a potent virucidal weapon that immobilizes the virus as well as inhibits viral fusion and thus entry into the host cells. Furthermore simulation of this mechanism *in vitro* might provide an interesting new therapeutic approach against microorganisms.

## Introduction

Despite recent advances in the understanding of HIV-1 infection, major scientific mysteries remain and HIV-1 infection is still a global challenge for mankind. Viruses exclusively depend on the host cellular machinery for their propagation and survival and therefore need to invade into a host cell.

Genome entry into the target cell involves for the enveloped HIV-1 several major steps. In most infections HIV-1 binds to a cell-surface receptor and co-receptors; subsequently, as binding is not sufficient for entry, the HIV-1 and the host cell membrane fuse.

Membrane fusogenicity is the result of an active virus-host cell interaction process that induces conformational changes within the viral envelope protein [1].

Structural rearrangements within gp120 allow the binding to co-receptors of the chemokine-receptor family, which aids in virus entry. HIV-1 uses two important co-receptors. The chemokine receptor CCR5 is the co-receptor utilized by macrophage- or M-tropic viruses. They are referred to as R5 viruses [2]. The chemokine receptor CXCR4 is the co-receptor for T-tropic or X4 viruses [3]. It is especially used by viruses appearing later in HIV-1 infection [4]. Dual-tropic HIV-1 strains use both co-receptors (R5X4 strains).

The triggering of fusion requires cleavage of two of the nine disulfide bonds of gp120 by the cell-surface protein disulfide-isomerase (PDI) [5]. Activation of fusogenicity can result from precise thiol/disulfide rearrangements mediated by either an endogenous redox autocatalytic isomerase or a cell-associated oxidoreductase [1]. In addition thiol/disulfide exchange in the HIV-1 envelope protein is a prerequisite for CXCR4-tropic HIV-1 envelope-mediated T-cell fusion [6].

To inhibit the contact between the envelope protein and its receptor and co-receptors might therefore be an important target for body defence.

The innate immune system is the first line of defence against invading pathogens. Entry of HIV-1 into the host initiates immediate responses to control virus propagation. Neutrophils are recruited to sites of viral infection and act as the first line of defence [7].

Myeloperoxidase (MPO), a heme enzyme released from azurophilic intracellular granules by activated neutrophils, is the most abundant protein in neutrophils [8].

At physiological concentrations chloride is the most likely substrate and hypochlorous acid (HOCl) has been shown to be a major end-product [9]. The pK_a_ values for of HOCl is 7.59, thus at physiological pHs, approximately equal concentration of HOCl and ^−^OCl will be present [10]. We use the term HOCl to describe this mixture.

Klebanoff and colleagues showed already in the beginning of the 90^th^ that the myeloperoxidase/HOCl system of polymorphonuclear leukocytes and mononuclear phagocytes has a virucidal effect against HIV-1 [11], [12]. *In vitro* the heme enzyme myeloperoxidase is very efficient in stopping HIV-1 propagation [13]. HOCl reacts with and kills microbes directly.

Myeloperoxidase can be released to the outside of the cell, where it attacks normal tissue [14]. Segal found that the majority of proteins that get oxidised by the myeloperoxidase hypohalide system surprisingly belong to the host not the invaders [15]. Plasma proteins consume the majority of HOCl. HOCl-mediated oxidation of the major plasma protein, albumin, was investigated by Pattison et al [16].

While there is no doubt that the myeloperoxidase system is capable of inactivating HIV-1 directly, it may have in addition an indirect mode of action.

As proteins and amino acids are highly abundant *in vivo* and react rapidly with HOCl, they are likely to be major targets for HOCl [17]. Exposure of proteins to HOCl can result in the loss of protein structure because of unfolding [18].

The finding that HOCl-altered proteins (we named them **i**mmune **de**fence-**a**ltered-**p**roteins, Idea-Ps) were amyloid-like and bound with high affinity to proteins offering free SH-groups and the recently published knowledge on the amyloid-like “Semen-derived Enhancer of Viral Infection” (SEVI) and the role of thiols in HIV-1 entry prompted us to study whether Idea-Ps are able to inhibit HIV-1 infection.

## Results

### 1. Immune defence-altered pooled plasma proteins (Idea-PP) bind with high affinity to HIV-1 gp120, its receptor CD4 and to PDI

**Figure 1:**
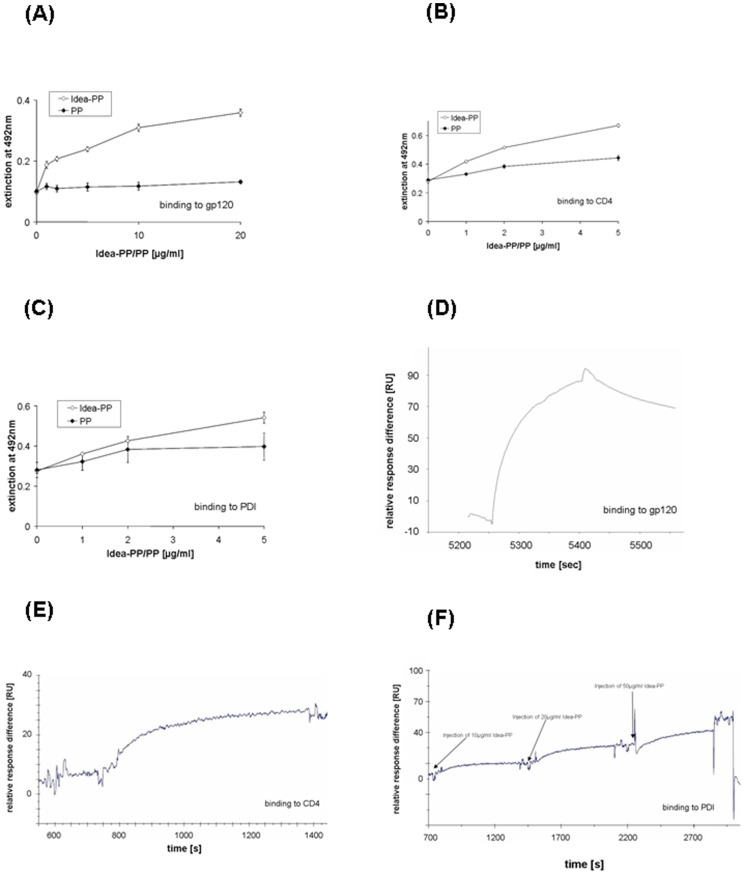
Binding of pooled plasma proteins (PP) and HOCl-altered pooled plasma proteins (Idea-PP) to gp120, CD4 and PDI. Binding of either plasma proteins (PP) or altered plasma proteins (Idea-PP) to purified HIV-1 gp120 (A), CD4 (B) or PDI (C), respectively was determined by specific ELISA. The mean extinction ± SD from three parallel samples is presented. Surface plasmon resonance sensorgrams of the binding of Idea-PP to gp 120 (Fig. 1 D), to CD4 (Fig. 1 E), and to PDI (Fig. 1 F) showed high affinity.

To investigate the consequences of protein modification by the myeloperoxidase/HOCl immune defence system pooled plasma proteins were isolated from human plasma, modified by HOCl to generate Idea-PP and subsequently tested for their capacity to bind to HIV-1 gp120, its receptor CD4 and to the PDI. As a control unaltered pooled plasma proteins (PP) were used for the binding studies. ELISA assays showed specific binding to gp120, CD4 as well as to the PDI for Idea-PP, whereas binding of the non-modified original material to GP120 and CD4 was not significant (Fig. 1A, B). Some binding of the non-modified original material to the PDI was observed, but the binding to the HOCl-altered material was significantly higher.

Binding was confirmed by surface plasmon resonance (SPR) spectrometry. SPR demonstrated that immobilized gp120, CD4 as well as PDI bind Idea-PP with very high affinity (Fig. 1D, E, F).

The sensorgrams could have been fitted to a Langmuir model using BiaEvaluation Software 3.1 (GE Healthcare) to gain quantitative data, but the off-rates were too slow to be reliably estimated by this method. The association phase of a BIAcore experiment is also affected by the dissociation rate. Inherent in the fitting method is in addition the assumption that the association and dissociation rates are true for a one-to-one interaction model. As Idea-PP is a mixture of proteins and several of them oligomerize and presumably present several binding sites, this model cannot be used and therefore no reliable quantitative data can be given.

In all control experiments using unmodified plasma protein the response units (RU) in SPR were below 10.

Thus Idea-PPs represent putative therapeutic agents to interfere with viral binding to and fusion with host cells.

### 2. Idea-PP inhibit cellular infection by HIV-1 IIIB

**Figure 2:**
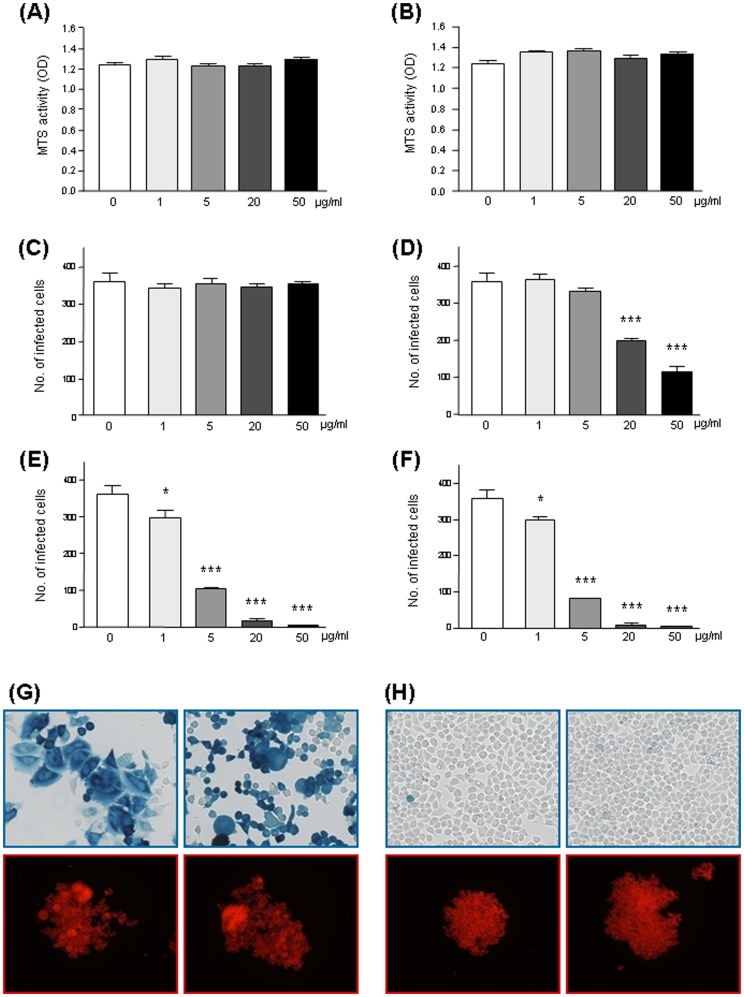
Effect of plasma proteins (PP) and HOCl-altered plasma proteins (Idea-PP) on HIV-1 infection. A putative toxicity of PP (A) or Idea-PP modified in a protein∶HOCl ratio of 1∶2000 (B) was controlled by measuring mitochondrial activity of HeLa-T4lacZ cells with MTS assay. Idea-PP is not toxic to host cells in the used concentrations. The optical density is presented as mean ± SD from three parallel samples. The X4-tropic HIV-1-isolate IIIB was pre-incubated for 1 h with purified plasma proteins, either unaltered (C) or modified by HOCl in a ratio PP∶HOCl of 1∶300 (D), 1∶1000 (E), or 1∶2000 (F) and given to HeLa-T4lacZ cells. The number of infected cells was quantified after 4d from four parallel samples (C; D, E; F). Microscopic view on HIV-1-infected HeLa-T4lacZ cells (G, H upper part) and M8166 monocytes (G, H lower part). HIV-1-IIIB was pre-incubated for 1 h either with PP (E) or with Idea-PP, modified with HOCl in a ratio 1∶2000 (F) and added to the cells; the cells were visualized after 4d (G, H).

In order to study the effect of Idea-PP on viral binding to and fusion with human cells pooled plasma proteins (PP) were altered to Idea-PP using different molar ratios of protein to HOCl; ratios of 1∶300, 1∶1000 and 1∶2000 were used for the experiments to evaluate the optimal procedure. Neither Idea-PP nor the control PP was toxic for the human cells in the used concentrations of up to 50 µg/ml since the mitochondrial activity was not decreased. (Fig. 2A, B)

The first assay to test the effect of Idea-PP on binding to and fusion with human cells was based on an adherent CD4+ HeLa cell line stably transfected with episomal vectors carrying the β-galactosidase gene under the control of the HIV-1 long terminal repeat (LTR) promoter. HIV-1 infection of these cells transactivates the LTR promoter inducing β-galactosidase production. Infected cells stained dark blue after the addition of the chromogenic substrate X-Gal. All tested Idea-PP pools reduced the number of HIV-1-infected cells in a dose dependent manner (Fig. 2D, E, F), whereas the non-modified original material had no effect (Fig. 2C). However the antiviral effect was more prominent when Idea-PP was produced with a molar ratio of protein to HOCl of 1∶1000 (Fig. 2E: IC90 0.5 µg/ml, IC50 3.5 µg/ml) compared to the material generated with a ratio 1∶300 (Fig. 2D: IC90 7 µg/ml, IC50 31 µg/ml). A higher ratio of 1∶2000 did not further increase the virus-inhibitory effect of Idea-PP (Fig. 2F: IC90 0.5 µg/ml, IC50 3.5 µg/ml).

Light microscopy visualized that nearly all host cells treated with the control pooled plasma protein (PP) were stained intensely blue, showing to be infected by HIV-1 IIIB (Fig. 2G upper row), while only sporadic host cells were infected by HIV-1 IIIB, when the virus was pre-incubated with Idea-PP (1∶1000) (Fig. 2H upper row).

HIV-1 IIIB is capable of inducing syncytia formation of infected host cells. Infected host cells fuse together induced by CD4-gp120 interaction on the plasma membrane to form large to giant cells with several nuclei. Inhibition of syncytia formation by Idea-PP was seen using M8166 cells, while pooled plasma protein control had no effect (Fig. 2G lower row for PP control versus Fig. 2H lower row for Idea-PP).

**Figure 3:**
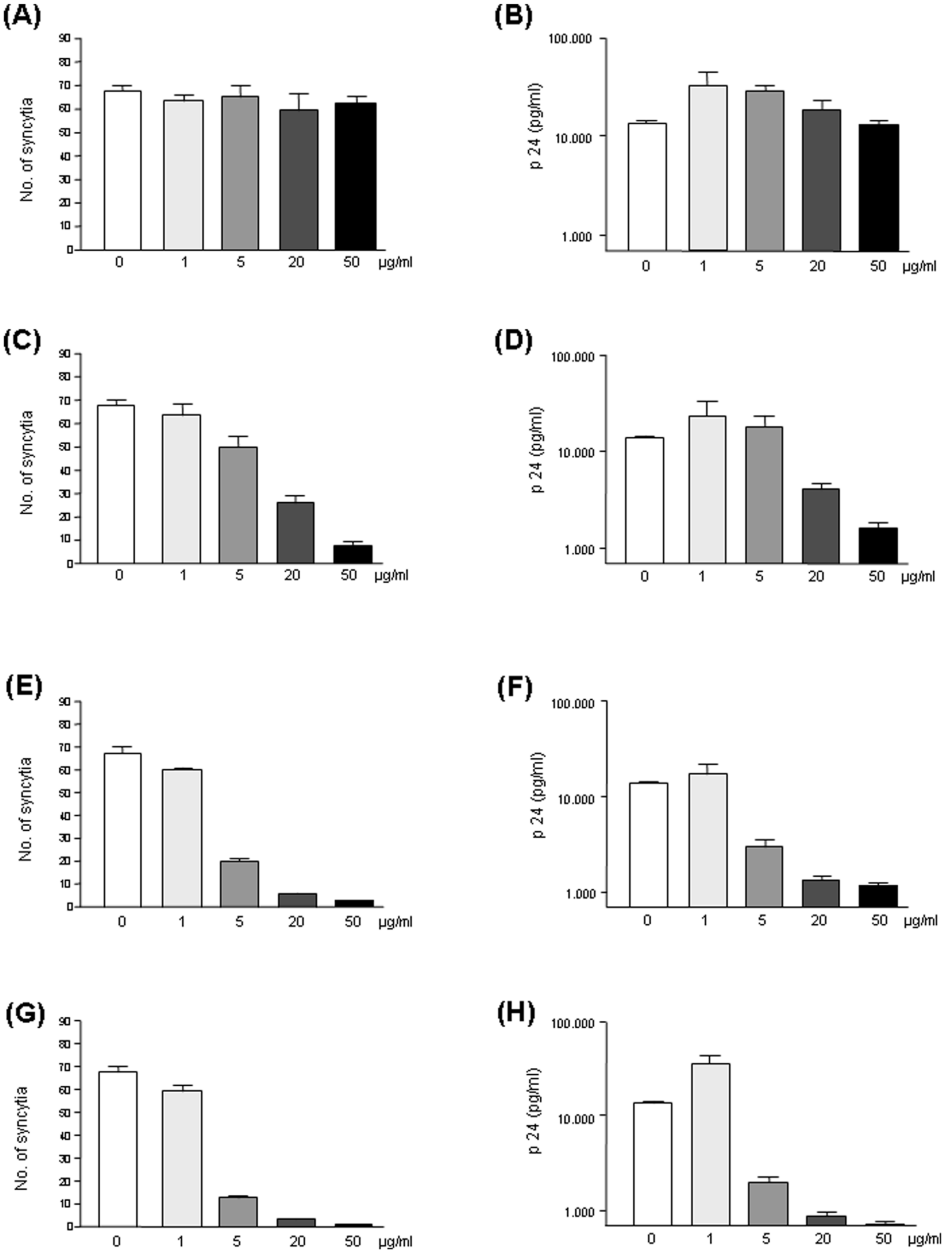
Effect of plasma proteins (PP) and HOCl-altered plasma proteins (Idea-PP) on HIV-1-induced syncytia formation and viral replication. The X4-tropic HIV-1-isolate IIIB was pre-incubated for 1 h with purified plasma proteins, either unaltered (A,B) or modified by HOCl in a ratio PP∶HOCl of 1∶300 (C,D), 1∶1000 (E,F), or 1∶2000 (G,H) and given to HeLa-T4lacZ cells. After 4d the number of syncytia (A,C,E,G) was quantified microscopically and the amount of p24 in the culture supernatant (B,D,F,H) was measured by ELISA. The values are given as mean ± SD from four parallel samples.

Pooled plasma protein control had in addition no influence on the formation of HIV-1-induced HeLaT4-lacZ syncytia (Fig. 3A). HIV-1 particles pre-incubated with increasing concentrations of PP induced not less syncytia as those treated with medium only. In contrast, the number of syncytia decreased, when the viral particles were incubated with Idea-PP generated with different molar ratios of protein∶HOCl (Fig. 3C, E, G). As seen in the previous experiment where the number of infected host cells was quantified, Idea-PP prepared with a ratio of 1∶1000 (protein∶HOCl) was more effective to interfere with syncytia formation than Idea-PP prepared with a ratio of 1∶300 (Fig. 3C: IC90 2 µg/ml, IC90 16 µg/ml, Fig. 3E: IC90 0.5 µg/ml, IC50 3.5 µg/ml). A higher ratio of 1∶2000 could not further increase the inhibitory effect of Idea-PP on virus induced syncytia formation (Fig. 3G: IC90 0.5 µg/ml, IC50 3.5 µg/ml).

Measuring the HIV-1 major capsid protein p24 antigen in cell culture supernatant is a long-established method for virus quantification. While unmodified pooled plasma proteins did not reduce the amount of p24 in the cell supernatant (Fig. 3B) Idea-PP decreased the p24 levels in a dose-dependent manner (Fig. 3D: IC90 11 µg/ml, IC50 30 µg/ml; Fig. 3F: IC90 2 µg/ml, IC50 5 µg/ml; Fig. 3H: IC90 2.5 µg/ml, IC50 4.5 µg/ml). These results correspond to those seen for the number of syncytia.

### 3. Idea-PP also inhibits other X4-tropic as well as R5X4-tropic HIV-1 strains

**Figure 4:**
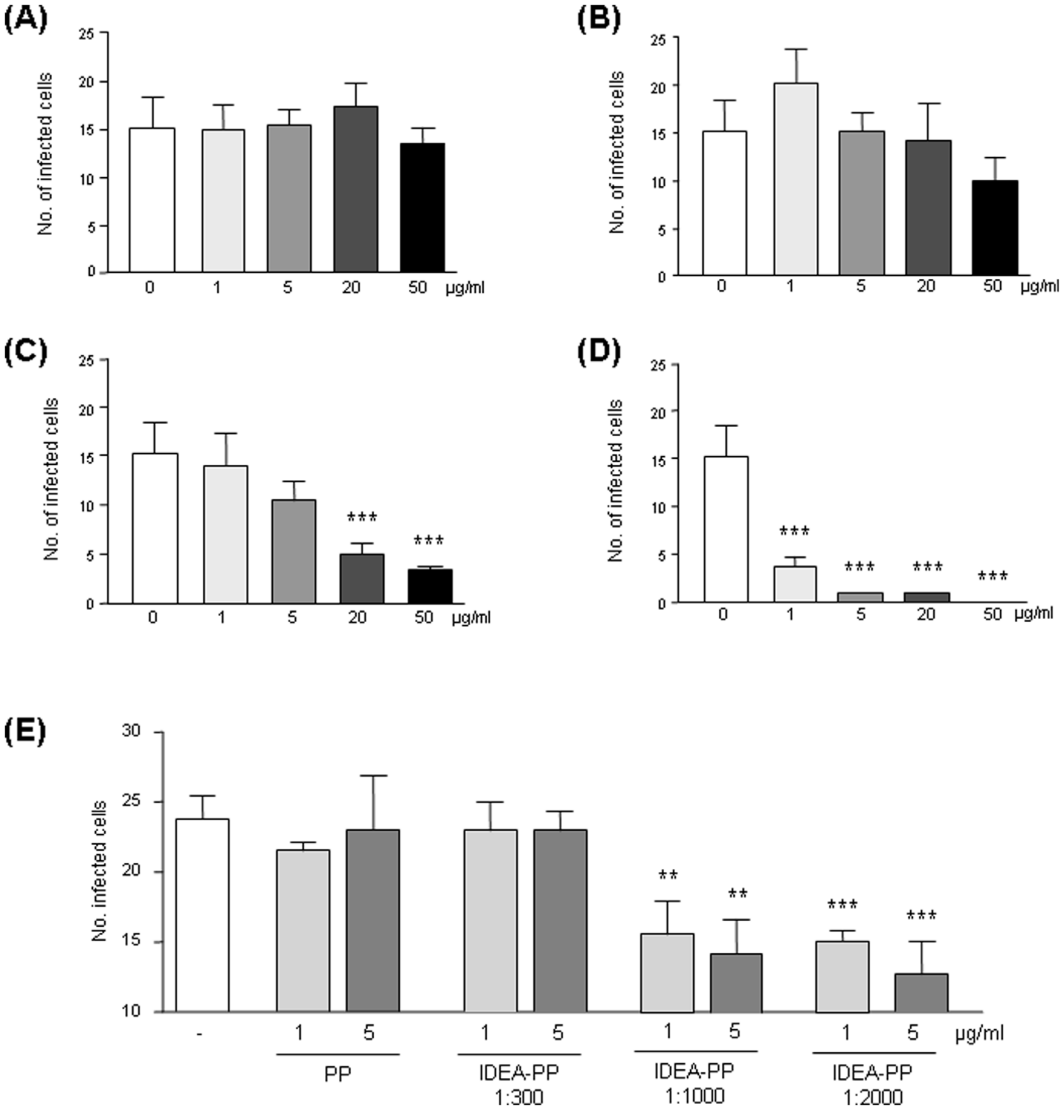
Effect of plasma proteins (PP) and HOCl-altered plasma proteins (Idea-PP) on cellular infection by different HIV-1 isolates. The X4-tropic HIV-1 isolate NL4-3 was pre-treated with the same substances and given to HeLaT4-lacZ cells (A). The number of infected cells was quantified after 4d, the values are given as mean ± SD from four parallel samples. The R5X4-tropic HIV-1 isolate 93BR020 (B–E) was pre-incubated for 1 h with purified plasma proteins, either unaltered (E) or modified by HOCl in a ratio PP∶HOCl of 1∶300 (D), 1∶1000 (B), or 1∶2000 (C) and given to HeLa-T4lacZ cells.

To answer the question whether inhibition of HIV-1 is strain-specific or represents a more general phenomenon, we tested the capacity of Idea-PP to inhibit other HIV-1 isolates than IIIB. Infection of HeLaT4-lacZ by another X4-tropic viral strain, NL4-3, was significantly reduced by pre-incubation of the HIV-1 particle with Idea-PP prepared with a molar ratio of protein∶HOCl of 1∶1000 (Fig. 4A).

Infection of HeLaT4-lacZ by the R5X4-tropic HIV-1 clone 93BR020 (subtype F), a primary isolate by WHO from a Brazilian patient in 1993, was inhibited by pre-incubation with Idea-PP prepared at molar ratios of protein∶HOCl of 1∶1000 (IC90 2 µg/ml, IC50 13 µg/ml or 1∶2000 (IC90 0.1 µg/ml, IC50 0.8 µg/ml) (Fig. 4B, C) in a dose-dependent manner. The effect of Idea-PP prepared with a molar ratio of protein∶HOCl of 1∶300 was less prominent (Fig. 4D) and control PP had no effect (Fig. 4E).

### 4. Isolated single immune defence-altered proteins (Idea-Ps) show antiviral activity

In a further set of experiments we evaluated purified plasma proteins before and after alteration with HOCl for their capacity to interfere with viral infectivity. For that reason we chose human serum albumin (HSA) as the most prominent plasma protein and antithrombin III (ATIII), as representatives for all plasma proteins, and compared the antiviral effect of their modified forms with that of the corresponding unmodified proteins.

**Figure 5:**
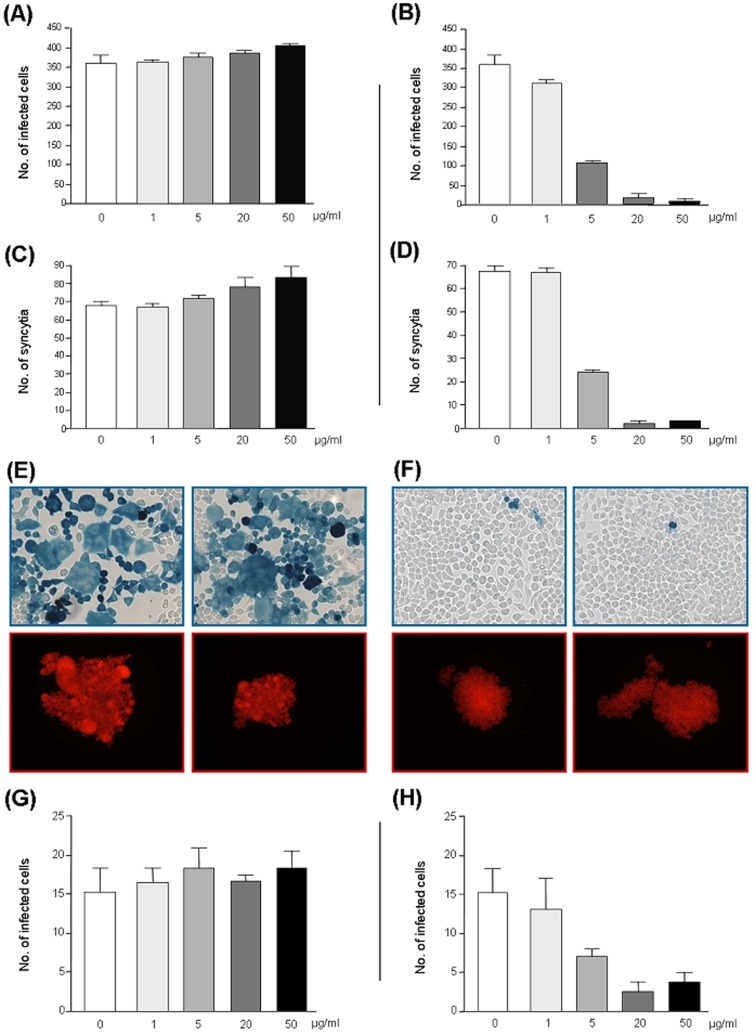
Effect of human serum albumin (HSA) and HOCl-altered HSA (Idea-HSA) on HIV-1 infection. The X4-tropic HIV-1-isolate IIIB was pre-incubated for 1 h with purified HSA, either unaltered (A,C) or modified by HOCl in a ratio HSA∶HOCl of 1∶1000 (B,D) and given to HeLa-T4lacZ cells. The number of infected cells (A,B) and the number of syncytia (C,D) was quantified after 4d from four parallel samples. (E,F) Microscopic view on HIV-1-infected HeLa-T4lacZ cells (E, F upper part) and M8166 monocytes (E, F lower part). HIV-1-IIIB was pre-incubated either with HSA (E) or with Idea-HSA (F) and added to the cells; the cells were visualized after 4d. The dualtropic HIV-1-strain 93BR020 was pre-incubated with either HSA (G) or with IDEA-HSA 1∶1000 (H). Numbers of infected cells after 4d are presented as mean ± SD from four parallel samples.

Inhibition of viral infection by Idea-HSA in comparison to HSA could be observed as reduction of infected cells (Fig. 5A, B; Fig. 5E upper row versus Fig. 5F upper row) (Fig. 5B: IC90 0.7 µg/ml, IC50 3.5 µg/ml) as well as a decrease in the number of syncytia (Fig. 5C, D; Fig. 5E lower row versus Fig. 5F lower row) (Fig. 5D: IC90 1.5 µg/ml, IC50 4.5 µg/ml).

The antiviral effect of Idea-HSA was not limited to X4-tropic isolate IIIB, since the dualtropic primary isolate 93BR030 was also inactivated by contact with Idea-HSA (Fig. 5G, H).

**Figure 6:**
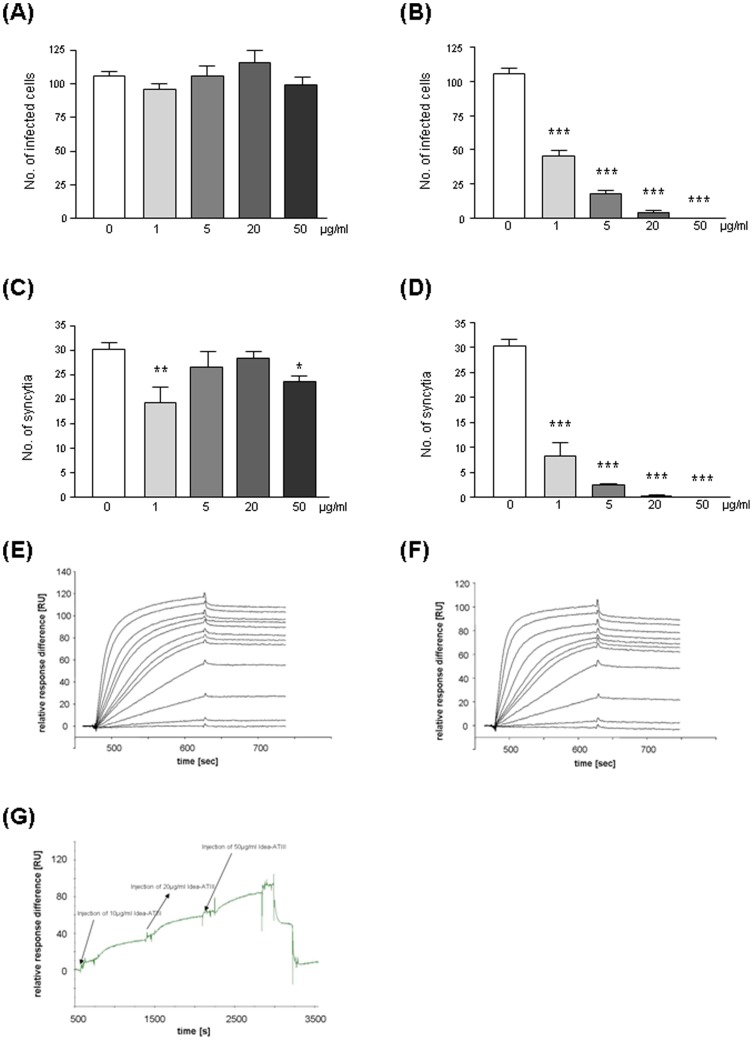
Effect of antithrombin III (ATIII) and HOCl-altered ATIII (Idea-ATIII) on HIV-1 infection. The X4-tropic HIV-1-isolate IIIB was pre-incubated for 1 h with purified ATIII, either unaltered (A,C) or modified by HOCl in a ratio 1∶1000 (B,D) and given to HeLa-T4lacZ cells. The number of infected cells (A,B) and the number of syncytia (C,D) per well was quantified microscopically after 4d. Numbers are presented as mean ± SD from four parallel samples. Surface plasmon resonance sensorgrams of the binding of Idea-ATIII to gp120 (E), to CD4 (F), and to PDI (G) showed high affinity.

To verify, that acquiring antiviral activity by modification with HOCl is not restricted to albumin, we included modified antithrombin III in our experiments. When HIV-1 was pre-incubated with increasing concentrations of Idea-ATIII the infectivity of the virus was highly decreased as shown by reduced number of infected HeLaT4-lacZ cells and lower amounts of syncytia (Fig. 6A: IC90 0.2 µg/ml, IC50 1 µg/ml; Fig. 6C: IC90 0.1 µg/ml, IC50 0.8 µg/ml). No effect was visible when the virus was incubated with unmodified ATIII (Fig. 6B, D). Surface Plasmon Resonance (SPR) was used to monitor the interactions in real time.

As seen with altered plasma protein mixture Idea-ATIII showed also high-affinity binding to gp120 (Fig. 6E), to CD4 (Fig. 6F) and to the PDI (Fig. 6G). Reliable on and off rates and dissociation rate constants k_d [s_^−1^_]_ can not be given, as the KD is dependent on both the association (on) and dissociation (off) rates of the compound and the off-rates were too slow to be meaningfully determined.

In addition to antithrombin III and HSA we tested fibrinogen, coagulation factor VIII and bovine serum albumin. All these proteins were successfully transformed into antiviral agents by alteration with HOCl.

### 5. Mechanism of antiviral activity by Idea-P

As the Idea-proteins used in this study tended to oligomerize and aggregate in contrast to the unmodified proteins, we tested whether they are partially unfolded or misfolded by the HOCl treatment and therefore amyloid-like. Coagulation factor XII (FXII), tissue plasminogen activator (tPA) and the chaperone BiP, all proteins capable to bind proteins with amyloid-like properties, bound all tested Idea-proteins (Idea-PP, Idea-HSA, Idea-ATIII) (figure S1) and HOCl treatment of proteins induced oligomerisation. In addition we previously made the observation that Idea-P bound reduced glutathione, GSH. To further clarify the mechanism how Idea-Ps exert their antiviral activity we tested therefore the hypothesis that the binding of Idea-P to gp120, CD4 and PDI, respectively, is at least partially thiol-mediated.

The inhibitor of free sulfhydryls that does not penetrate the membrane, 5,5′-Dithio-bis(2-nitrobenzoic-acid) (DTNB), was used to examine the role of ecto-sulfhydryls in the interaction of Idea-P with gp120, CD4 and PDI.

**Figure 7:**
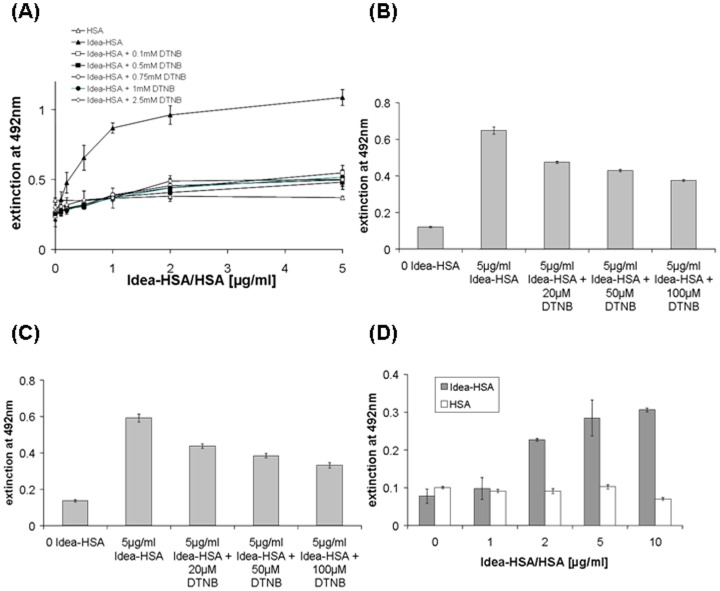
Binding of HIV-1-gp120, CD4 and PDI to HOCl-altered HSA (Idea-HSA) in the presence of cell impermeable thiol blocker DTNB and detection of FDP-lysine on Idea-HSA. The HIV-1 envelope protein gp120 (A), CD4 (B) and PDI (C) were pre-incubated with increasing concentrations of the thiol reagent DTNB. Binding of Idea-HSA (1 µg/ml) was quantified by a specific ELISA. The extinction is given as mean ± SD from three parallel samples. Idea-HSA (D) and HSA were immobilized and the binding of the FDP-lysine specific monoclonal antibody (clone 5F6) was tested by ELISA. The extinction is given as mean ± SD from three parallel samples.

The viral envelope protein gp120, its receptor CD4 or PDI, respectively, were pre-incubated with increasing concentrations of the thiol blocker DTNB. Binding of Idea-HSA to gp120, to CD4 as well as to PDI were inhibited by DTNB (Fig. 7A, B, C). In addition the binding of Idea-PP, Idea-ATIII and other HOCl-altered proteins was inhibited by the direct thiol blocker (data not shown).

HOCl-treatment of proteins can lead to the formation of α, β-unsaturated aldehydes. These can react with lysine groups to form the SH-binding form N^ε^-(3-formyl-3,4-dehydropiperidino) lysine (FDP-lysine) group. Therefore we tested whether Idea-Ps with antiviral activity contain FDP-lysine groups.

A specific monoclonal antibody against FDP-lysine bound specifically and dose-dependently to all our Idea-P preparations. Fig. 7 D shows the binding of the anti FDP-lysine monoclonal antibody to Idea-HSA, but not to HSA.

## Discussion

Neutrophils mediate important human innate immune responses to infection. Host defence against microbial invaders is initiated by the respiratory burst, in which nicotinamide adenine dinucleotide phosphate (NADPH) oxidase generates vast amounts of superoxide anions. The enzyme myeloperoxidase (MPO) uses hydrogen peroxide and chloride as a co-substrate to generate hypochlorous acid, a potent antimicrobial agent. While killing of microorganisms was previously believed to be accomplished directly by free oxygen radicals and by oxidized halides, the group of Segal showed that the killing activity of neutrophils is mediated mainly through activation of proteases by K^+^ flux [19], [20]. Neutrophils cooperate with extracellular agents as well as other immune cells to optimally kill and degrade invading microbes [21]. Intense activation by microbial or inflammatory stimuli triggers neutrophil degranulation. It involves granule translocation to the plasma membrane followed by exocytosis, a tightly regulated process [22]. Excessive degranulation of azurophilic, primary granules from neutrophils is a central feature in numerous inflammatory disorders, like cardiovascular disease [23].

As proteins and amino acids are highly abundant *in vivo* and react rapidly with HOCl, they are likely to be major targets for HOCl [16], [17]. Oxidation by myeloperoxidase-derived HOCl can cause both a loss of function of protease inhibitors and lysozyme [24], but also a gain of function has been reported [25]. Most proteins that become altered by the myeloperoxidase/HOCl system do not belong to the invading microorganisms, but to the host [20]. We speculated, that HOCl-altered host proteins might have a role in host defence. Therefore plasma proteins were modified by the myeloperoxidase/HOCl system. The altered protein mixture still bound to immobilized GSH after very carefully removal of HOCl. As HIV-1-gp120 [26], host cell CD4 [27], and the PDI [28] all present ecto-sulfhydryls essential for HIV-1 entry, it was examined whether Idea-PP is able to bind to these proteins. Using ELISA and plasmon resonance technique specific and high affinity binding of Idea-PP to gp120, CD4 as well as to the PDI was observed.

However, the off-rates were very slow (<5×10^−5^ s^−1^), and analysis of slow dissociation rates using Biacore is really challenging [29]. As HOCl altered proteins oligomerize and several binding sites may be presented on Idea-Ps to gp120, CD4 as well as the PDI avidity effects from multivalent binding may obscure the intrinsic thermodynamic affinities of the single binding sites. The sensorgrams indicate very tight binding but giving binding parameters and calculate affinities by the surface plasmon resonance (SPR) data would not be meaningful.

Binding of Idea-PP to gp120, CD4 and PDI prompted us to speculate, that the body might make use of Idea-PP as viral entry inhibitors. MTS cell viability assay to determine potential cytotoxicity of Idea-PP documented that neither control PP nor Idea-PP were toxic to host cells and therefore were used in subsequent testing on HIV-1 infectivity.

The effect of Idea-PP was thereon examined using three different, independent test systems. Idea-PP, but not control PP, inhibited in a dose-dependent manner cellular infection by HIV-1 IIIB as documented by a semi-quantitative assay based on the transactivation of the HIV-1 LTR promoter. IdeaPP manufactured with a ratio of 1∶300 (PP∶HOCl) was less effective than Idea-PP produced with a ratio of 1∶1000. Using more HOCl for Idea-PP preparation did not further intensify the effect of the Idea-PP. The morphology of all Idea-PP or PP treated cells looked perfectly normal.

HIV-1 infection *in vitro* can induce syncytia formation, giant multinuclear cells that are generated by cell fusion [30]. Idea-PP but not PP inhibited HIV-1 IIIB-induced syncytia formation and interfered with infectivity in HeLaT4-lacZ cells in a dose-dependent manner. Again Idea-PP prepared with a ratio of 1∶1000 (PP∶HOCl) was most effective. Furthermore viral propagation in the cell culture could nearly completely be abolished by pre-incubation of HIV-1 particles with Idea-PP, but not with PP control as shown with p24 assay. The culture-adapted HIV-1 IIIB strain is X4-tropic and infects mainly T-cells. Therefore further experiments were performed with another X4-tropic viral strain, NL4-3, and a primary isolate from a patient the R5X4 tropic HIV-1 clone 93BR020 (subtype F9). Inhibition of infectivity, syncytia formation and viral propagation showed that the anti HIV-1-effect of Idea-PP is more general at least to X4-tropic HIV-1 strains.

The next step was to elucidate, whether the myeloperoxidase/HOCl system can transform also isolated single proteins into antiviral weapons. As albumin is the most abundant plasma protein human serum albumin was altered with HOCl to Idea-HSA. As seen with Idea-PP mixture Idea-HSA reduced in a dose-dependent manner the number of cells infected by the X4-tropic isolate HIV-1 IIIB as well as the dual tropic isolate 93BR030. To test whether the transformation into an antiviral weapon is human albumin specific, several other plasma proteins were treated with HOCl. Antithrombin III, coagulation factor VIII and bovine serum albumin were altered to produce Idea-ATIII, Idea-FVIII, Idea-BSA. As shown for Idea-HSA, each one of the other altered plasma proteins reduced the number of HIV-1 infected cells and lowered the amounts of HIV-1 induced syncytia. For Idea-ATIII and Idea-HSA binding to gp120, CD4 and to the PDI with high affinity, was demonstrated [31], [32].

Naturally occurring fragments of the prostatic acidic phosphatase (PAP) form amyloid fibrils termed Semen-derived Enhancer of Virus Infection (SEVI), capture HIV-1 particles and promote their attachment to target cells, thereby enhancing the infectious virus titre by several orders of magnitude [33]. These data show clearly that the HIV-1 binds to amyloidogenic proteins.

Winter et al demonstrated that low molar ratios of HOCl to protein cause oxidative protein unfolding [34]. All the HOCl modified proteins used in this work showed amyloid-like properties, as they oligomerized, tended to aggregate and bound to the general amyloid recognizing proteins tPA, FXII and the chaperone BiP. Other proteins, shown to interact with HIV-1 and to inhibit HIV-1 infection, are also known to have amyloid-like properties. One of these proteins is thrombospondin-1 [35]. Thrombospondin-1 aggegated the virus and inhibited viral propagation by binding to the env-protein [36]. Later Thrombospondin-1 was demonstrated to share structural properties with amyloid proteins [37]. The alpha-defensins, Human Neutrophil Peptide-1–3, exhibited anti-HIV-1 activity on at least two levels: directly inactivating virus particles; and affecting the ability of target CD4 cells to replicate the virus [38]. Human alpha defensins 1–3 bind to tPA [39], oligomerize in the presence of a membrane and bind to the chaperone protein BiP [40]. In that way they seem to act like amyloid-like proteins. Human neutrophil alpha defensins 1–3 are in addition able to induce amyloid-like structures in adhesion proteins like fibrinogen, von Willebrand factor, fibronectin and thrombospondin-1 [40].

### As amyloid-like proteins bind to HIV-1, oligomerize, and built aggregates and nets, they will probably immobilize the virus!

Neutrophils contribute to pathogen clearance by producing neutrophil extracellular traps (NETs). Akong-Moore et al [41] very recently studied NETs formation and found that, while exogenous addition of H_2_O_2_ and HOCl stimulated NETosis, only exogenous HOCl could rescue NETosis in the setting of MPO inhibition. The myeloperoxidase associated with neutrophil extracellular traps is active [42], and therefore likely producing Idea-Ps. Recently Saitoh et al [43] showed that NETs in addition to bacteria and fungi also capture the human immunodeficiency virus. Idea-proteins are very special amyloid-like proteins, as they have a second mode of action to interfere with HIV-1-infection. Meshes built by chromatin strands and by amyloid-like Idea-Ps may support each other.

Idea-proteins bind to immobilized GSH and binding of Idea-proteins to gp120, CD4 and to the PDI was inhibited by DTNB, showing, that Idea-P likely is interacting with the ecto-sulfhydryls of gp120, CD4 and PDI. Treatment of plasma protein with the myeloperoxidase/HOCl system was shown to lead to the conversion of L-threonine into 2-hydroxypropanal and its dehydration product, acrolein (2-propenal), an extremely reactive α, β-unsaturated aldehyde [44]. Aldehyde production was also mimicked by hypochlorous acid (HOCl) in the absence of the enzyme. In a murine model of acute myocardial infarction, the myeloperoxidase/HOCl system was found to be a major source of acrolein [45]. The α,β-unsaturated aldehyde acrolein can form bis-adducts with lysine residues, which are called FDP-lysines [46]. The electrophilic α,β-unsaturated carbonyl moiety is retained in FDP-lysine, allowing it to undergo a nucleophilic addition of thiols [47]. Protein-conjugated FDP-lysine was detected by the FDP-lysine specific antibody F56 [46]. Using antibody F56 we documented that Idea-HSA, but not the unmodified mother protein HSA, contained FDP-lysine residues. As Idea-Ps binding to gp120, CD4 and PDI is inhibited by DTNB and Idea-Ps clearly contain accessible chemical groups that react eagerly with free SH groups, it is likely that SH-groups are at least in part involved in the interaction between Idea-Ps and HIV-1. FDP-lysine groups have been shown to bind covalently to free SH groups of the protein glyceraldehyde-3-phosphate dehydrogenase [47]. Probably FDP-lysine groups in Idea-Ps will also link Idea-Ps to its targets on HIV-1 in a covalent manner. The very slow off rates seen in the sensorgrams of the plasmon resonance data support this interpretation.

**Figure 8:**
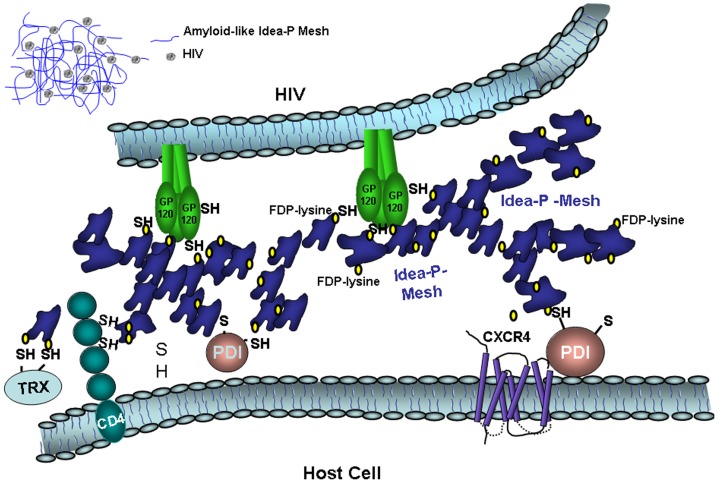
Hypothetical mechanism of blocking HIV-1-entry by Idea-P. We postulate that the myeloperoxidase/HOCl system, in addition to its other roles in host defence, works as a mechanism to form HIV-1 inhibitors by forming amyloid-like proteins that immobilizes the virus and by the generation of FDP-lysine residues on these proteins that attack ecto-sulfhydryls in gp120, CD4 and in the PDI. Idea-proteins have an amyloid-like structure and bind with high affinity to proteins and regents offering free thiol groups. As these amyloid-like proteins bind to HIV-1, oligomerize, and built aggregates and nets, they will probably immobilize the virus and interfere with its binding to the cell surface chaperone GRP78/BiP. High affinity binding was shown to gp120, CD4 as well as to PDI. Thiol-blocker DTNB inhibited the binding of Idea-P to gp120, CD4 and PDI showing that the binding was SH-dependent. Other proteins involved in HIV-1 entry that offer free SH-groups are thioredoxin, a 12-kD redox protein, necessary for disulfide exchange in domain 2 of CD4, and the protein disulfide isomerase (PDI), essential for reduction of gp120 disulfide bonds, which triggers the major conformational changes in gp120 and gp41 required for virus entry. We hypothesize that Idea-P can inhibit on several levels HIV-1 entry by immobilization and by blocking free thiols.

In conclusion we postulate that the myeloperoxidase/HOCl system, in addition to its other roles in host defence, works as a mechanism to form HIV-1 entry inhibitors by forming amyloid-like proteins that immobilizes the virus and by the generation of FDP-lysine residues on these proteins that attack ecto-sulfhydryls in gp120, CD4 and in the PDI (Fig. 8).

The question, why there are not enough Idea-Ps to combat HIV-1 invasion, might be answered by the observation that there is significant impairment of neutrophil function in advanced HIV-1 infection and by the recent observation that the HIV-1 counteracts neutrophil response [41]. In addition to neutropenia, a variety of qualitative defects in neutrophil function has been described in HIV-1 infection [48]–[50].

Other microorganisms depend on an amyloid coat to attach to host cells [51] and on active thiols for entry into the host cell as well. The coxsackievirus A9 and dengue fever virus serotype 2 use the amyloid recognizing chaperone BiP as a co-receptor for virus internalization [52], [53]. Ryser et al described that HIV-1 entry into host cells can be inhibited by bacitracin [28]. Bacitracin is usually considered to be a specific inhibitor of PDI activity. Recently, however, bacitracin has been shown to have in addition significant effects on both no catalyzed protein folding and on other molecular chaperones. Especially bacitracin significantly inhibits the chaperone activity of BiP, the general binding protein for amyloid-like proteins [54]. As Idea-proteins bind to BiP and the known HIV-1 entry inhibitor bacitracin inhibits BiP, it is likely that in addition to the chaperone PDI, the chaperone GRP78/BiP, a principal sensor for amyloidogenic proteins, is involved in HIV-1-infection. Involvement of cell surface BiP would also explain the enhancement of HIV-1 infectivity by Semen-derived Enhancer of Virus Infection (SEVI). As other viruses have been shown to use BiP for internalisation [55], [56] Idea-proteins might also be useful as inhibitors of other virus infections. Jain et al observed that blocking the production of free thiols by thiol-disulfide exchange inhibitors of the fusion protein (F) of the Newcastle disease virus (NDV) the F protein inhibited membrane fusion required for viral entry and cell-cell fusion [57]. The role of thiol-disulfide exchange in host cell entry/invasion is not restricted to viruses. According to Conant et al *Chlamydia* attachment to mammalian cells requires protein disulfide isomerase [58], and disulfide oxidoreductase activity of *Shigella flexneri* is required for invasion of epithelial cells [59]. The protein disulfide isomerase of the parasite *Neospora canium* is involved in tachyzoite-host cell interaction [60].The PDI was even identified to play an important role in cancer cell (glioma) invasion [61]. Therefore our newly discovered neutrophil function might be of general relevance to path physiology.

## Materials and Methods

### Ethic Statement

Ethics approval for this study was obtained from the Innsbruck Medical University, Austria.

The PBMCs were separated from a blood donation. Written informed consent was obtained from the participating, anonymized blood donors by the Central Institute for Blood Transfusion and Immunological Department, Innsbruck, Austria.

### Cell lines and culture

The human epithelial-like cell line HeLa-T4 PBK-LTRLac are adherent CD4-positive cells, stably transfected with an episomal vector carrying the Escherichia coli β-galactosidase gene under the control of the HIV-1 long terminal repeat (LTR) promoter [62]. The cell line was purchased from the Programme EVA Centre for AIDS Reagents (Hertfordshire, UK) and cultured in DMEM medium (Life Technologies, Vienna, Austria) supplemented with 10% fetal calf serum (FCS; BioWhittaker, Verviers, Belgium), penicillin/streptomycin (100 U/ml and 100 µg/ml, respectively), 2 mM L-glutamine (Life Technologies), 1 mg/ml geneticin (selection for CD4 antigen; Sigma) and 100 µg/ml hygromycin B (selection for pBK-LTRLac episome; Sigma).

The T-lymphoblastoid cell line M8166 was cultivated in RPMI 1640 medium (Life Technologies) supplemented with 10% FCS, penicillin/streptomycin and 2 mM L-glutamine.

### Virus propagation and purification

The X4-tropic HIV-1 strain IIIB (subtype B) was from the MRC [63], [64] and grown for propagation in the cell line M8166. The R5X4-tropic HIV-1 clone 93BR020 (subtype F) was isolated by WHO from a brazilean patient in 1993 [65] and propagated in human peripheral blood mononuclear cells (PBMC), isolated from healthy human blood donors. The X4-tropic viral strain NL4-3 was purchased from AIDS Research and Reference Reagent Program, NIAID, NIH [64]. This virus is a full-length, replication and infection competent chimera with the 5′ fragment derived from proviral NY5 and the 3′ fragment from proviral LAV.

Virus-containing cell culture supernatants were harvested 3 days after infection and filtered to remove cell debris. Viral particles were purified by ultracentrifugation at 20,000 *g* for 1 h; the pellet was resuspended in RPMI medium and frozen at −80°C.

Viral yield was determined by p24 antigen ELISA developed at the Institute of Applied Microbiology (Vienna, Austria). For that assay the monoclonal antibodies 37G12 and Mo1 were used, a kind gift from H. Katinger (Institute for Applied Microbiology). Briefly, microplates were coated overnight with the first monoclonal antibody and washed three times with PBS/0.1% Tween20 (Serva, Heidelberg, Germany). Dilution series of viral stocks were applied on the plate, together with the second antibody conjugated with biotin, for 1 h. The plates were consequently incubated with streptavidin-β-galactosidase (Roche, Wien, Austria) for 30 min. The amount of bound p24 was quantified by adding resorufin-β-D-galactopyranoside substrate solution (Sigma, St. Louis, USA) and measuring the optical density at 550 nm using an ELISA reader. The p24 assay was calibrated using baculovirus-derived recombinant p24 as a standard.

### Preparation of Idea-P

Normal purified human proteins were transformed into the antiviral form (Idea-Ps) by adding HOCl, to mimic the action of the myeloperoxidase/H_2_O_2_/HOCl system, in a molar ratio (protein∶HOCl 1∶100 to 1.2000) as given in the results. To alter complex plasma protein compositions the estimated averaged molecular weight of 70 kD for the proteins was taken as a basis. In brief, freshly prepared HOCl was added to the protein in phosphate buffered salt solution (PBS, pH 7.2) and the mixture was incubated for 30 min at RT. Remaining HOCl was separated by gel-filtration using sephadex G25 PD10 columns (GE Healthcare). To transform protein mixture from human plasma into immune defence-altered plasma protein (Idea-PP), proteins were precipitated from human plasma with ammonium sulfate, dialysed to remove the ammonium sulfate and then modified directly with freshly prepared HOCl.

### Idea-P toxicity tests

To examine a potential toxicity of Idea-Ps, the HeLa cells were incubated with increasing amounts of the substances and the mitochondrial activity was analyzed using a commercially available test kit according to manufacturer's instruction (Promega, Madison, USA). After 4 days MTS reagent was added to the cells and its conversion by active mitochondrial enzymes was measured in an ELISA-reader by determining the optical density at dual wavelengths at 492 nm and 620 nm.

### Virus infection and neutralization by Idea-Ps

The HeLa CD4+ cell line was infected with the different viruses at a concentration of 40–400 ng/ml p24. For this purpose the virus was pre-incubated either with medium or with different concentrations of Idea-Ps for 1 h at room temperature. The mixture was given to the cells for 24 h, followed by removal of the cell culture supernatant, intensive washing and addition of fresh medium either with or without Idea-Ps. To quantify viral infection and propagation three different tests were applied after 4 days. First, the number of formed syncytia per well was quantified microscopically. Second, secretion of progenitor virus in the culture supernatant was examined using the p24 assay described above. Third, the number of infected cells can be investigated by addition of the chromogenic substrate X-Gal, since viral infection transactivates the LTR promoter inducing β-galactosidase production. For this purpose the cells were washed with PBS followed by fixing with 0.5% glutaraldehyde for 15 min at room temperature. Afterwards this solution was replaced by PBS containing 200 µg/ml X-Gal, 3 mM potassium ferricyanide, 3 mM potassium ferrocyanide, 1.3 mM MgCl_2_ (pH 7.4). After incubation for 2–6 h at 37°C blue foci were counted microscopically.

M8166 cells were incubated with 0.25 to 3 ng/ml virus pre-incubated with medium with or without Idea-Ps as described above. Viral infection and propagation was quantified by counting the syncytia in the microscope.

### Binding of Idea-P to GP120, CD4 as well as to PDI quantified by ELISA

Full length and glycosylated recombinant gp120 LAV (IIIb), gp120 LAV and recombinant CD4 were purchased from Protein Sciences Corporation (Meriden, CT, USA). Protein Disulfide Isomerase (PDI) was from Takara (Otsu, Japan). Binding of the different Idea-Ps to gp120 IIIB, CD4 as well as to the PDI were demonstrated in a standard ELISA. In brief, the bottom of the wells of NUNC MaxiSorp™ polystyrene 96 well ELISA plates were coated with gp120, CD4 or PDI, respectively (100 ng/well in 100 µl coating buffer) over night at 4°C. The selected coating and blocking buffers were 15 mM disodium carbonate and 25 mM sodium hydrogen carbonate buffer, pH 9.6, and 0.25% gelatine solution with phosphate-buffered saline solution +0.05% Tween20 (PBS-T), respectively. The wells were blocked for 1 h at RT. Plates were washed 4 times with 300 µl washing buffer (PBS-T) per well and all residual washing buffer was removed. 100 µl Idea-P samples or unaltered mother proteins were added to appropriate wells and incubated at 37°C for 1 hour. The plates were washed thoroughly and an antibody against HSA, Fibrinogen or Antithrombin III (DAKO, Hamburg, Germany) was added. Plates were washed again and a secondary peroxidase labeled antibody was added to detect the primary antibody in concentrations recommended by the manufacturer. 100 µl ATBS substrate was given to each well, the plates were wrapped to shield from light, incubated at room temperature and the absorbance was read at OD_492._ Experiments run at least in triplicate.

### Binding of Idea-P to gp120, to CD4 as well as to PDI using Plasmone Resonance

Surface plasmon resonance (SPR) biosensors enable rapid and label free sensing of biomolecular interactions. The SPR phenomenon occurs when polarized light, under conditions of total internal reflection, strikes an electrically conducting gold layer. A Biacore 2000 and a Biacore 3000 system (BIACORE AB, Sweden) were used. HIV-1-gp120, CD4 or PDI, respectively were immobilized on a C5 sensor chip using amine coupling kit (BIACORE AB). Conditions were as follows: running buffer 25 mM Tris, 10 mM NaCl, pH 7.4, 1 mM CaCl_2_, 1 mM MgCl_2_, 0.005% surfactant P-20; coupling buffer 10 mM NaAc pH 7.0; HIV-1-gp120 20 µl (10 µg/ml), immobilized amount: 1158RU; CD4 30 µl (6.3 µg/ml), immobilized amount 568RU; PDI 20 µl (10 µg/ml), immobilized amount 1396 RU.

After the immobilisation free amino groups on the chip surface were quenched with ethanolamine and the binding of Idea-Ps was investigated. As an example for other Idea-Ps the experimental procedure for Idea-ATIII is given in detail. 50 µl Idea-ATIII was injected in three different concentrations at a flow rate of 20 µl/min. The protein solutions were diluted with sample buffer. With increasing Idea-ATIII amounts the resulting signals increased. Overlap sensorgrams, which show binding of Idea-ATIII to immobilized HIV-1-gp120, CD4 and PDI and subsequent dissociation were created. All the sensorgrams were corrected by subtracting the signal from reference flow cell. The non-altered original proteins and protein mixtures were used as controls.

### Inhibition of Idea-P binding to gp120, CD4 or PDI, respectively by SH-reactive substances

Dithiobis-nitrobenzoic acid (DTNB, Sigma, Taufkirchen, Germany) (100 µl per well, 0.1 to 2.5 mM in PBS, pH 7.2) or para-chloromercuriphenyl sulfonate (pCMPS, Sigma, Taufkirchen, Germany) (100 µl, 10 to 300 µM in PBS, pH 7.2) were added to immobilized gp120, immobilized CD4 or immobilized PDI in wells of an ELISA-plate for 15 minutes, and unbound thiol-reagent was washed away thoroughly. Afterwards the plates were processed as described above to measure binding of Idea-P to gp120, CD4 or PDI, respectively.

### Amyloid-like structure of Idea-PP, Idea-HSA, Idea ATIII

As Maas et al have disclosed, that tPA, FXII as well as the chaperone BiP are capable of binding proteins with amyloid modules [66], binding of Idea-PP, Idea-HSA, Idea-ATII to all three general amyloid recognizing tools was studied by an ELISA.

Recombinant tPA (Actilyse®) and coagulation factor FXII were purchased from Boehringer Ingelheim (Ingelheim, Germany) or Haemochrom. Diagnostica. (Essen, Germany), respectively. Recombinant human BiP was from Stressmarq™ Biosciences Inc. (Victoria, Canada).

Binding of the different Idea-Ps to tPA, FXII as well as to BiP were demonstrated in a standard ELISA. In brief, the bottom of the wells of NUNC MaxiSorp™ polystyrene 96 well ELISA plates were coated with tPa, FXII or BiP, respectively (100 ng/well in 100 µl coating buffer) over night at 4°C. The selected coating and blocking buffers were 15 mM disodium carbonate and 25 mM sodium hydrogen carbonate buffer, pH 9.6, and 0.25% gelatine solution with phosphate-buffered saline solution +0.05% Tween20 (PBS-T), respectively. The wells were blocked for 1 h at RT. The plates were washed 4 times with 300 µl washing buffer (PBS-T) per well and all residual washing buffer was removed. 100 µl Idea-PP, Idea-HSA, Idea-ATIII samples or unaltered mother proteins were added to appropriate wells and incubated at 37°C for 1 hour. The plates were washed thoroughly and a specific antibody was added. Plates were washed again and a secondary peroxidase labeled antibody was added to detect the primary antibody in concentrations recommended by the manufacturer. 100 µl ATBS substrate was given to each well, the plates were wrapped to shield from light, incubated at room temperature and the absorbance was read at OD_492._ Experiments run at least in triplicate.

## Supporting Information

Figure S1 **Binding of Idea-PP, Idea-HSA or Idea-ATIII to immobilised coagulation factor XII, tissue plasminogen activator or the chaperone BiP.** The binding to coagulation factor FXII (A, B, C), to tPA (D, E, F) or to BiP (G, H, I) of altered plasma proteins (Idea-PP, A, D, G), altered human serum albumin (Idea-has, B, E, H) or altered antithrombin III (Idea-ATIII, C,F,I) was determined by specific ELISA. The mean extinction ± SD from three parallel samples is presented.
(TIF)

## References

[1] Fenouillet E, Barbouche R, Jones IM (2007) Cell entry by enveloped viruses: redox considerations for HIV and SARS-coronavirus. Antioxid Redox Signal 9 (8) 1009–34. 17567241 10.1089/ars.2007.1639

[2] Berger EA, Murphy PM, Farber JM (1999) Chemokine receptors as HIV coreceptors: roles in viral entry, tropism, and disease. Annu Rev Immunol 17: 657–700. 10358771 10.1146/annurev.immunol.17.1.657

[3] Simmons G, Reeves JD, Hibbitts S, Stine JT, Gray PW, et al (2000) Co-receptor use by HIV and inhibition of HIV infection by chemokine receptor ligands. Immunol Rev 177: 112–26. 11138769 10.1034/j.1600-065x.2000.17719.x

[4] Connor RI, Sheridan KE, Ceradini D, Choe S, Landau NR (1997) Change in Coreceptor Use Correlates with Disease Progression in HIV–Infected Individuals. J Exp Med 185 (4) 621–8. 9034141 10.1084/jem.185.4.621 PMC2196142

[5] Barbouche R, Lortat-Jacob H, Jones IM, Fenouillet E (2005) Glycosaminoglycans and protein disulfide isomerase-mediated reduction of HIV Env. Mol Pharmacol 67 (4) 1111–8. 15644496 10.1124/mol.104.008276

[6] Markovic I, Stantchev TS, Fields KH, Tiffany LJ, Tomiç M, et al (2004) Thiol/disulfide exchange is a prerequisite for CXCR4-tropic HIV envelope-mediated T-cell fusion during viral entry. Blood 103 (5) 1586–94. 14592831 10.1182/blood-2003-05-1390

[7] Jenne CN, Wong CH, Zemp FJ, McDonald B, Rahman MM, et al (2013) Neutrophils recruited to sites of infection protect from virus challenge by releasing neutrophil extracellular traps. Cell Host Microbe 13 (2) 169–80. 23414757 10.1016/j.chom.2013.01.005

[8] Schultz J, Kaminker K (1962) Myeloperoxidase of leucocyte of normal human blood. I. Content and localization. Arch Biochem Biophys 96 (3) 465–467. 13909511 10.1016/0003-9861(62)90321-1

[9] Pullar JM, Vissers MC, Winterbourn CC (2000) Living with a Killer: The Effects of Hypochlorous Acid on Mammalian. Cells 50 (4–5) 259–266. 10.1080/713803731 11327319

[10] Morris JC (1966) The acid ionization constant of HOCl from 5°C to 35°C. J Phys Chem 70: 3798–3806.

[11] Klebanoff SJ, Coombs RW (1992) Viricidal effect of polymorphonuclear leukocytes on HIV: role of the myeloperoxidase system. J Clin Invest 89: 2014–2017. 1318327 10.1172/JCI115810 PMC295907

[12] Chase MJ, Klebanoff SJ (1992) Viricidal effect of stimulated human mononuclear phagocytes on human immunodeficiency virus type 1. Proc Natl Acad Sci USA 89: 5582–5585. 1319066 10.1073/pnas.89.12.5582 PMC49336

[13] Chochola J, Yamaguchi Y, Moguilevsky N, Bollen A, Strosberg AD, et al (1994) Virucidal effect of myeloperoxidase on human immunodeficiency virus type 1- infected T cells. Microb Agents Chemother 38 (5) 969–972. 10.1128/aac.38.5.969 PMC188135 8067778

[14] Klebanoff SJ (2005) Myeloperoxidase: friend and foe. J Leukoc Biol 77 (5) 598–625. 15689384 10.1189/jlb.1204697

[15] Segal AW, Garcia RC, Harper AM, Banga JP (1983) Iodination by stimulated human neutrophils. Studies on its stoichiometry, subcellular localization and relevance to microbial killing. Biochem J 210 (1) 215–25. 6303312 10.1042/bj2100215 PMC1154208

[16] Pattison DI, Hawkins CL, Davies MJ (2009) What are the plasma targets of the oxidant hypochlorous acid? A kinetic modelling approach. Chem Res Toxicol 22 (5) 807–17. 19326902 10.1021/tx800372d

[17] Pattison DI, Hawkins CL, Davies MJ (2007) Hypochlorous acid-mediated protein oxidation: how important are chloramine transfer reactions and protein tertiary structure? Biochemistry 46 (34) 9853–64. 17676767 10.1021/bi7008294

[18] Chapman ALP, Winterbourn CC, Brennan SO, Jordan TW, Kettle AJ (2003) Characterization of non-covalent oligomers of proteins treated with hypochlorous acid. Biochem J 375: 33–40. 12852783 10.1042/BJ20030685 PMC1223668

[19] Reeves EP, Lu H, Jacobs HL, Messina CG, Bolsover S, et al (2002) Killing activity of neutrophils is mediated through activation of proteases by K+ flux. Nature 416 (6878) 291–7. 11907569 10.1038/416291a

[20] Segal AW (2005) How neutrophils kill microbes. Annu Rev Immunol 23: 197–223. 15771570 10.1146/annurev.immunol.23.021704.115653 PMC2092448

[21] Nauseef WM (2007) How human neutrophils kill and degrade microbes: an integrated view. Immunol Rev 219: 88–102. 17850484 10.1111/j.1600-065X.2007.00550.x

[22] Lacy P, Eitzen G (2008) Control of granule exocytosis in neutrophils. Front Biosci 13: 5559–70. 18508605 10.2741/3099

[23] Nicholls SJ, Hazen SL (2005) Myeloperoxidase and cardiovascular disease. Arterioscler Thromb Vasc Biol 25 (6) 1102–11. 15790935 10.1161/01.ATV.0000163262.83456.6d

[24] Hawkins CL, Davies MJ (2005) Inactivation of protease inhibitors and lysozyme by hypochlorous acid: role of side-chain oxidation and protein unfolding in loss of biological function. Chem Res Toxicol 18 (10) 1600–10. 16533025 10.1021/tx050207b

[25] Undurti A, Huang Y, Lupica JA, Smith JD, DiDonato JA, et al (2009) Modification of high density lipoprotein by myeloperoxidase generates a pro-inflammatory particle. J Biol Chem 284 (45) 30825–35. 19726691 10.1074/jbc.M109.047605 PMC2781481

[26] Kornbluth RS (2004) HIV envelope becomes unhinged by PDI for entry. Blood 103: 1567.

[27] Matthias LJ, Yam PT, Jiang XM, Vandegraaff N, Li P, et al (2002) Disulfide exchange in domain 2 of CD4 is required for entry of HIV. Nat Immunol 3 (8) 727–32. 12089508 10.1038/ni815

[28] Ryser HJ, Levy EM, Mandel R, DiSciullo GJ (1994) Inhibition of human immunodeficiency virus infection by agents that interfere with thiol-disulfide interchange upon virus-receptor interaction. Proc Natl Acad Sci U S A 91 (10) 4559–63. 8183947 10.1073/pnas.91.10.4559 PMC43825

[29] Nieba L, Krebber A, Pluckthun A (1996) Competition BIAcore for measuring true affinities: large differences from values determined from binding kinetics. Analytical biochemistry 234: 155–165. 8714593 10.1006/abio.1996.0067

[30] Dalgleish AG, Beverley PC, Clapham PR, Crawford DH, Greaves MF, et al (1984) The CD4 (T4) antigen is an essential component of the receptor for the AIDS retrovirus. Nature 312 (5996) 763–7. 6096719 10.1038/312763a0

[31] Kehrel BE, Brodde MF (2002) Medicament containing activated antithrombin III. Patent application. WO 2002/022150 A2; priority date 2000-09-12.

[32] Kehrel BE, Brodde MF (2004) Oxidized proteins, their biological activity, and therapeutic and diagnostic measures, which are derived from the active mechanism, from the use of these proteins or from the inhibition thereof. Patent application. US 2004/0047861; priority date 2000-10-20.

[33] Münch J, Rücker E, Ständker L, Adermann K, Goffinet C, et al (2007) Semen-derived amyloid fibrils drastically enhance HIV infection. Cell 131 (6) 1059–71. 18083097 10.1016/j.cell.2007.10.014

[34] Winter J, Ilbert M, Graf PC, Ozcelik D, Jakob U (2008) Bleach activates a redox-regulated chaperone by oxidative protein unfolding. Cell 135 (4) 691–701. 19013278 10.1016/j.cell.2008.09.024 PMC2606091

[35] Crombie R, Silverstein RL, MacLow C, Pearce SF, Nachman RL, et al (1998) Identification of a CD36-related thrombospondin 1-binding domain in HIV envelope glycoprotein gp120: relationship to HIV-specific inhibitory factors in human saliva. J Exp Med 187 (1) 25–35. 9419208 10.1084/jem.187.1.25 PMC2199189

[36] Crombie R (2000) Mechanism of Thrombospondin-1 Anti-HIV Activity. AIDS Patient Care STDS 14 (4) 211–4. 10806640 10.1089/108729100317821

[37] McDonald JF, Dimitry JM, Frazier WA (2003) An amyloid-like C-terminal domain of thrombospondin-1 displays CD47 agonist activity requiring both VVM motifs. Biochemistry 42 (33) 10001–11. 12924949 10.1021/bi0341408

[38] Mackewicz CE, Yuan J, Tran P, Diaz L, Mack E, et al (2003) alpha-Defensins can have anti-HIV activity but are not CD8 cell anti-HIV factors. AIDS 17 (14) F23–32. 14502030 10.1097/00002030-200309260-00001

[39] Higazi AA, Ganz T, Kariko K, Cines DB (1996) Defensin modulates tissue-type plasminogen activator and plasminogen binding to fibrin and endothelial cells. J Biol Chem 271 (30) 17650–5. 8663495 10.1074/jbc.271.30.17650

[40] Horn M, Bertling A, Brodde MF, Müller A, Roth J, et al (2012) Human neutrophil alpha defensins induce formation of fibrinogen and thrombospondin-1 amyloid-like structures and activate platelets via GPIIbIIIa. J Thromb Haemostas 10: 647–661. 10.1111/j.1538-7836.2012.04640.x 22268819

[41] Akong-Moore K, Chow OA, von Köckritz-Blickwede M, Nizet V (2012) Influences of chloride and hypochlorite on neutrophil extracellular trap formation. PLoS One 7 (8) e42984. 22912772 10.1371/journal.pone.0042984 PMC3418225

[42] Parker H, Albrett AM, Kettle AJ, Winterbourn CC (2012) Myeloperoxidase associated with neutrophil extracellular traps is active and mediates bacterial killing in the presence of hydrogen peroxide. J Leukoc Biol 91 (3) 369–76. 22131345 10.1189/jlb.0711387

[43] Saitoh T, Komano J, Saitoh Y, Misawa T, Takahama M, et al (2012) Neutrophil extracellular traps mediate a host defense response to human immunodeficiency virus-1. Cell Host Microbe 19;12 (1) 109–16. 10.1016/j.chom.2012.05.015 22817992

[44] Anderson MM, Hazen SL, Hsu FF, Heinecke JW (1997) Human neutrophils employ the myeloperoxidase-hydrogen peroxide-chloride system to convert hydroxy-amino acids into glycolaldehyde, 2-hydroxypropanal, and acrolein. A mechanism for the generation of highly reactive alpha-hydroxy and alpha,beta-unsaturated aldehydes by phagocytes at sites of inflammation. J Clin Invest 99 (3) 424–32. 9022075 10.1172/JCI119176 PMC507815

[45] Vasilyev N, Williams T, Brennan ML, Unzek S, Zhou X, et al (2005) Myeloperoxidase-generated oxidants modulate left ventricular remodeling but not infarct size after myocardial infarction. Circulation 112 (18) 2812–20. 16267254 10.1161/CIRCULATIONAHA.105.542340

[46] Uchida K, Kanematsu M, Morimitsu Y, Osawa T, Noguchi N, et al (1998) Acrolein is a product of lipid peroxidation reaction. Formation of free acrolein and its conjugate with lysine residues in oxidized low density lipoproteins. J Biol Chem 273 (26) 16058–66. 9632657 10.1074/jbc.273.26.16058

[47] Furuhata A, Nakamura M, Osawa T, Uchida K (2002) Thiolation of protein-bound carcinogenic aldehyde. An electrophilic acrolein-lysine adduct that covalently binds to thiols. J Biol Chem 277: 27919–27926. 12032148 10.1074/jbc.M202794200

[48] Ellis M, Gupta S, Galant S, Hakim S, VandeVen C, et al (1988) Impaired neutrophil function in patients with AIDS or AIDS-related complex: a comprehensive evaluation. J Infect Dis 158 (6) 1268–76. 3058816 10.1093/infdis/158.6.1268

[49] Pitrak DL, Bak PM, DeMarais P, Novak RM, Andersen BR (1993) Depressed neutrophil superoxide production in human immunodeficiency virus infection. J Infect Dis 167: 1406–1410. 8388903 10.1093/infdis/167.6.1406

[50] Gabrilovich D, Ivanova L, Serebrovskaya L, Shepeleva G, Pokrovsky V (1994) Clinical significance of neutrophil functional activity in HIV infection. cand J Infect Dis 26 (1) 41–7. 10.3109/00365549409008589 8191239

[51] Gebbink MF, Claessen D, Bouma B, Dijkhuizen L, Wösten HA (2005) Amyloids–a functional coat for microorganisms. Nat Rev Microbiol 3 (4) 333–41. 15806095 10.1038/nrmicro1127

[52] Triantafilou K, Fradelizi D, Wilson K, Triantafilou M (2002) GRP78, a coreceptor for coxsackievirus A9, interacts with major histocompatibility complex class I molecules which mediate virus internalization. J Virol 76 (2) 633–43. 11752154 10.1128/JVI.76.2.633-643.2002 PMC136810

[53] Upanan S, Kuadkitkan A, Smith DR (2008) Identification of dengue virus binding proteins using affinity chromatography. J Virol Methods 151 (2) 325–8. 18562018 10.1016/j.jviromet.2008.05.001

[54] Karala AR, Ruddock LW (2010) Bacitracin is not a specific inhibitor of protein disulfide isomerase. FEBS Journal 277: 2454–2462. 20477872 10.1111/j.1742-4658.2010.07660.x

[55] Honda T, Horie M, Daito T, Ikuta K, Tomonaga K (2009) Molecular Chaperone BiP Interacts with Borna Disease Virus Glycoprotein at the Cell Surface. J of Virology 83: 12622–12625. 19776128 10.1128/JVI.01201-09 PMC2786760

[56] Jindadamrongwech S, Thepparit C, Smith DR (2010) Identification of GRP 78 (BiP) as a liver cell expressed receptor element for dengue virus serotype 2. Arch Virol 149: 915–927. 10.1007/s00705-003-0263-x 15098107

[57] Jain S, McGinnes LW, Morrison TG (2007) Thiol/disulfide exchange is required for membrane fusion directed by the Newcastle disease virus fusion protein. J Virol 81 (5) 2328–39. 17151113 10.1128/JVI.01940-06 PMC1865930

[58] Conant CG, Stephens RS (2007) Chlamydia attachment to mammalian cells requires protein disulfide isomerase. Cell Microbiol 9 (1) 222–32. 16925789 10.1111/j.1462-5822.2006.00783.x

[59] Watarai M, Tobe T, Yoshikawa M, Sasakawa C (1995) Disulfide oxidoreductase activity of Shigella flexneri is required for release of Ipa proteins and invasion of epithelial cells. Proc Natl Acad Sci U S A 92 (11) 4927–31. 7761426 10.1073/pnas.92.11.4927 PMC41820

[60] Naguleswaran A, Alaeddine F, Guionaud C, Vonlaufen N, Sonda S, et al (2005) Neospora caninum protein disulfide isomerase is involved in tachyzoite-host cell interaction. Int J Parasitol 35 (13) 1459–72. 16129440 10.1016/j.ijpara.2005.06.006

[61] Goplen D, Wang J, Enger PØ, Tysnes BB, Terzis AJ, et al (2006) Protein disulfide isomerase expression is related to the invasive properties of malignant glioma. Cancer Res 66 (20) 9895–902. 17047051 10.1158/0008-5472.CAN-05-4589

[62] Akrigg A, Wilkinson GW, Angliss S, Greenaway PJ (1991) HIV indicator cell lines. AIDS 5 (2) 153–8. 1903260 10.1097/00002030-199102000-00004

[63] Popovic M, Flomenberg N, Volkman DJ, Mann D, Fauci AS, et al (1984) Alteration of T-cell functions by infection with HTLV-I or HTLV-II. Science 1984 Oct 26;226 (4673) 459–62. 10.1126/science.6093248 6093248

[64] Cheng-Mayer C, Levy JA (1988) Distinct biological and serological properties of human immunodeficiency viruses from the brain. Ann Neurol 23 (Suppl) S58–61. 3258140 10.1002/ana.410230716

[65] Gao F, Robertson DL, Carruthers CD, Morrison SG, Jian B, et al (1998) A Comprehensive Panel of Near-Full-Length Clones and Reference Sequences for Non-Subtype B Isolates of Human Immunodeficiency Virus Type 1. J Virol 72 (7) 5680–5698. 9621027 10.1128/jvi.72.7.5680-5698.1998 PMC110237

[66] Maas C, Schiks B, Strangi RD, Hackeng TM, Bouma BN, et al (2008) Identification of fibronectin type I domains as amyloid-binding modules on tissue-type plasminogen activator and three homologs. Amyloid 15 (3) 166–80. 18925455 10.1080/13506120802193498

