# Supplementary figures and images for: Neutrophils Turn Plasma Proteins into Weapons against HIV-1
Source: PLoS One. 2013 Jun 26;8(6):e66073. doi: 10.1371/journal.pone.0066073 (PMC3694086; doi:10.1371/journal.pone.0066073)

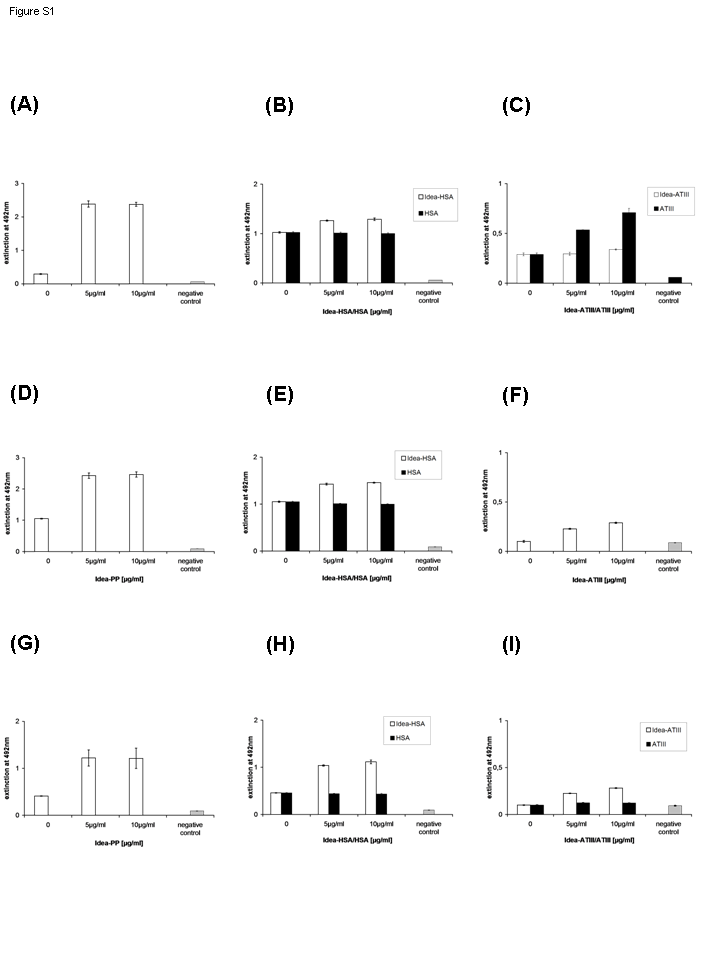

Supplement: Figure S1 — Binding of Idea-PP, Idea-HSA or Idea-ATIII to immobilised coagulation factor XII, tissue plasminogen activator or the chaperone BiP. The binding to coagulation factor FXII (A, B, C), to tPA (D, E, F) or to BiP (G, H, I) of altered plasma proteins (Idea-PP, A, D, G), altered human serum albumin (Idea-has, B, E, H) or altered antithrombin III (Idea-ATIII, C,F,I) was determined by specific ELISA. The mean extinction ± SD from three parallel samples is presented. (TIF) [file pone.0066073.s001.tif]
